# Development of A Low-Cost FPGA-Based Measurement System for Real-Time Processing of Acoustic Emission Data: Proof of Concept Using Control of Pulsed Laser Ablation in Liquids

**DOI:** 10.3390/s18061775

**Published:** 2018-06-01

**Authors:** Sebastian F. Wirtz, Adauto P. A. Cunha, Marc Labusch, Galina Marzun, Stephan Barcikowski, Dirk Söffker

**Affiliations:** 1 Chair of Dynamics and Control, University of Duisburg-Essen, 47057 Duisburg, Germany; adauto.cunha@uni-due.de (A.P.A.C.); soeffker@uni-due.de (D.S.); 2 Center for Nanointegration Duisburg-Essen (CENIDE), Nano Energy Technic Center (NETZ), Technical Chemistry I, University of Duisburg-Essen, 47057 Duisburg, Germany; marc.labusch@hs-niederrhein.de (M.L.); galina.marzun@uni-due.de (G.M.); stephan.barcikowski@uni-due.de (S.B.); 3 Institute for Coatings and Surface Chemistry (ILOC), Hochschule Niederrhein University of Applied Sciences, 47798 Krefeld, Germany

**Keywords:** acoustic emission, structural health monitoring, real-time signal processing, FPGA, embedded linux, wavelet transform, pulsed laser ablation in liquids, nanoparticles

## Abstract

Today, the demand for continuous monitoring of valuable or safety critical equipment is increasing in many industrial applications due to safety and economical requirements. Therefore, reliable in-situ measurement techniques are required for instance in Structural Health Monitoring (SHM) as well as process monitoring and control. Here, current challenges are related to the processing of sensor data with a high data rate and low latency. In particular, measurement and analyses of Acoustic Emission (AE) are widely used for passive, in-situ inspection. Advantages of AE are related to its sensitivity to different micro-mechanical mechanisms on the material level. However, online processing of AE waveforms is computationally demanding. The related equipment is typically bulky, expensive, and not well suited for permanent installation. The contribution of this paper is the development of a Field Programmable Gate Array (FPGA)-based measurement system using ZedBoard devlopment kit with Zynq-7000 system on chip for embedded implementation of suitable online processing algorithms. This platform comprises a dual-core Advanced Reduced Instruction Set Computer Machine (ARM) architecture running a Linux operating system and FPGA fabric. A FPGA-based hardware implementation of the discrete wavelet transform is realized to accelerate processing the AE measurements. Key features of the system are low cost, small form factor, and low energy consumption, which makes it suitable to serve as field-deployed measurement and control device. For verification of the functionality, a novel automatically realized adjustment of the working distance during pulsed laser ablation in liquids is established as an example. A sample rate of 5 MHz is achieved at 16 bit resolution.

## 1. Introduction

In many industries, the need for optimal use of equipment and resources is driving the development of new technologies. For example, Pulsed Laser Ablation in Liquids (PLAL) is a process for synthesis of nanoparticles from different materials. However, placement of the target material at a suitable Working Distance (WD) close to the focal point of the laser is crucial to achieve high productivity of PLAL. Zhu et al. [1] established a correlation between material ablation rate and audible acoustic waves (sound) using wideband microphone. This idea is used in this paper to implement an automatic positioning algorithm to improve productivity of PLAL. Different to [1], a surface mounted piezoelectric sensor is used to record Acoustic Emission (AE). Furthermore, a newly developed Field Programmable Gate Array (FPGA)-based measurement system is introduced, which is suitable for online processing of AE signals using Discrete Wavelet Transform (DWT) for noise reduction or feature extraction.

In general, AE refers to the phenomenon of transient elastic stress waves, which emerge from local sources [2]. Using highly sensitive measurement equipment and suitable signal processing, these waveforms can be detected and used to distinguish different source mechanisms. Key advantage is the sensitivity to damage on a material level including different micro-mechanical mechanisms e.g., crack nucleation and propagation [3], material removal [4], and wear [5]. In the field of SHM, applications of AE for in-situ measurements are manifold. Typically, SHM is understood as “the process of implementing a damage identification strategy” [6]. In this context, damage denotes an arbitrary change causing deviations from an initial state that have adverse effect on the performance of a system [6]. However, sensors can not measure damages directly and therefore suitable feature extraction and interpretation are required to provide statements regarding the current state of a system [7]. Mba [8] provided a review regarding AE for monitoring of rotating machinery such as gears and bearings. It was pointed out that AE is most mature in the field of bearing monitoring. However, it was also mentioned that effects of different operating conditions have to be further investigated to fully take advantage of the potential for SHM. Van Hecke et al. [9] developed a methodology to process AE signals tailored to improve diagnostic performance for low-speed bearings, which are typical for e.g., wind turbines. Baccar and Söffker [5] identified characteristic frequencies related to different wear mechanisms of wear resistant plates in friction contact and proposed a methodology for monitoring of tribological systems.

Besides SHM, the related technologies offer new opportunities for process monitoring and control. Here, reliable in-situ measurements are required to determine the current state of a process [10]. For example, Lee et al. [11] investigated AE for in-situ monitoring of precision manufacturing processes. Maia et al. [12] applied AE to monitor the condition of tools in turning operations. Here, adhesive and abrasive wear could be distinguished according to the frequency content of AE signals [12]. Moreover, Svecko et al. [13] used AE on an injection molding machine to detect damages of engraving tools during the manufacturing process. Furthermore, a review of AE related to chemical processes is provided by Boyd and Varley [10]. In particular, bubble formation in gas-liquid dispersions, transport processes, and chemical reactions are considered. It was pointed out that besides its applications to process monitoring, AE is ideally suited for the use in control systems [10].

During PLAL, high energy laser pulses with a duration on the order of several nano seconds are used to ignite a plasma on the surface of the target material. Furthermore, the high energy laser pulses cause formation of a cavitation bubble at the interface between the target material and the liquid. Nanoparticles are formed due to condensation in the gas phase of the cavitation bubble and are dispersed in the liquid after collapse [14]. Compared to chemical synthesis of nanoparticles, this process leads to a particularly clean product due to confinement in liquid environment e.g., water. The productivity (ablated mass per unit time) of PLAL depends strongly on the position of the target with respect to the focal point of the laser [15]. Thus, a suitable WD between the target and the laser is crucial. However, adjusting the WD manually is difficult and time consuming because direct measurement of the ablated mass requires disassembly of the test rig. Therefore, automatic adjustment of the WD based on in-situ measurements of the productivity is desirable. A typical method to measure the productivity in-situ is Ultraviolet/Visible (UV/VIS) spectroscopy. However, the use of this method for process monitoring and control is limited to a small concentration range. Furthermore, there is a dependence on the material, the particle size, and the particle shape. Therefore, AE measurements are used in this paper for automatic adjustment of the WD.

Ultrasonic sensing techniques including AE [2], Guided Waves [16], or Electromechanical Impedance (EMI) method [17] as well as sensing techniques based on optoelectronic principles [18,19,20,21] generate raw data which require complex signal processing at high data rates. By leveraging data parallelism of FPGA-based hardware architectures, the related signal processing algorithms can be implemented efficiently. In particular, AE is challenging due to several reasons. The wide frequency range of AE of up to 1 MHz necessitates high sampling rates. Furthermore, Kaphle et al. [22] identified the discrimination between damage related signals and spurious noises as a major challenge in AE monitoring. Traditionally, heuristically defined rules to extract parameters from AE waveforms are used to characterize the underlying source mechanism. In practice, this approach is widely used due to its simplicity and reduction of acquired data [22]. However, information regarding the underlying source mechanisms is limited using this approach. Therefore, in research the focus has moved towards advanced signal processing methods such as joint time-frequency domain transformations. Different methods including Short Time Fourier Transform (STFT), Hilbert Huang Transform (HHT), and Wavelet Transform (WT) are compared by Hamdi et al. [23]. It was concluded that suitability of STFT for decomposition of AE signals is limited due to fixed time-frequency resolution for a given window size. Also, it was pointed out that HHT is most suitable to decompose transient AE signals, whereas WT provides more flexibility in the analysis e.g., by choosing a suitable basis function. Furthermore, it is important to note that in contrast to HHT the Discrete Wavelet Transform (DWT) can be implemented for real-time use. Therefore, DWT is excessively used in context of AE for different purposes including feature extraction, denoising, and onset detection. For example, Marec et al. [24] used WT and C-means clustering to distinguish between different micro-mechanical fracture mechanisms in composite material. Moreover, Pomponi et al. [25] used thresholding of DWT coefficients for onset detection of AE transients in noisy measurements.

Especially when AE-based monitoring is based on information within higher frequency regimes [5], current challenges for measuring AE are related to signal processing under time constraints, storage, and accessibility of the data. Therefore, in this paper the development of an AE measurement system using low-cost FPGA-based platform, which is suitable for embedded implementation, is addressed. A FPGA-based implementation of the DWT, which is suitable for real-time use, is realized to accelerate processing of AE measurements. Furthermore, a wide range of I/O-interfaces such as Ethernet and USB are readily available to ensure data accessibility. Key features of the system are low cost, small form factor, and low energy consumption, which makes it suitable for field-deployed devices. For verification of the functionality, automatic adjustment of the WD during PLAL is established as an example.

The remainder of this paper is structured as follows. In Section 2, the experimental test rig for the PLAL is described. Furthermore, the developed AE measurement system is presented in detail. In particular, FPGA-based implementation of DWT, which is used to remove noise from the measurements, is addressed. In Section 3, experimental results are presented. Final remarks are given in Section 4.

## 2. Materials and Methods

Subsequently, the PLAL test rig used during the experimental study and the newly developed FPGA-based measurement system are described. Furthermore, particular emphasis is placed on the real-time implementation of the DWT, which can be used for denoising and feature extraction.

### 2.1. Laser Ablation Test Rig

**Figure 1:**
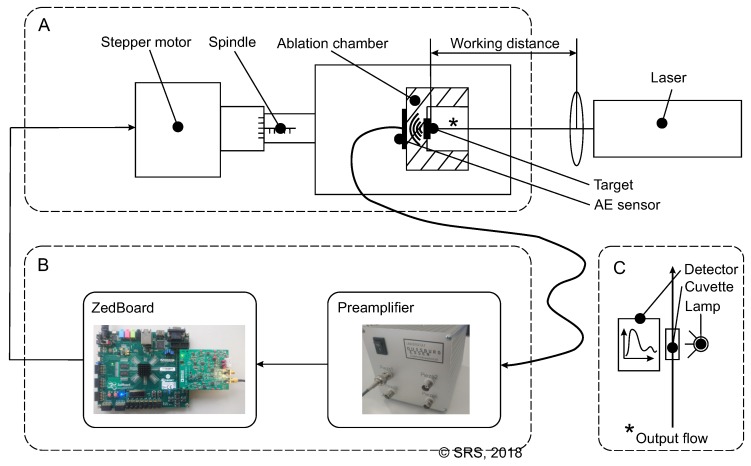
Illustration of the experimental setup: (**A**) Process plant; (**B**) Signal processing and control; (**C**) UV/VIS measurement.

The experimental setup in which the FPGA system is implemented is illustrated in Figure 1. The ablation chamber is constructed so that the target can be placed in a fixed position on a translatory precision stage, which is driven by a stepper motor to adjust the WD. As target material, gold and copper sheet metal (purity: 99.99 %) of 0.5 mm and 1 mm thickness are used, respectively. Furthermore, Milli-Q ultra pure water is used as liquid. Continuous water flow through the ablation chamber at a fixed flow rate of 50 mL/min is realized using plunger pump Ismatec RHP 100994.

The AE related to the PLAL is recorded by a piezoelectric element, which is permanently mounted on the back side of the ablation chamber. A preamplifier is used for signal conditioning before the AE signal is digitized and processed on the ZedBoard (Figure 1). Here, control input of the stepper motor is calculated based on the AE measurements. Additionally, as a reference UV/VIS spectroscopy is used to monitor the nanoparticle concentration in the liquid by conducting the output flow of the ablation chamber through a cuvette (1 cm path length). According to Rehbock et al. [26], extinction in the UV/VIS spectrum at a wavelength of 380 nm is proportional to the nanoparticle concentration. A detailed summary of the equipment used during the experiments is provided in Table 1.

### 2.2. System Overview

Subsequently, the newly developed FPGA-based AE measurement and control system is described. As a computational platform the ZedBoard (xc7z020clg484-1) is chosen. This is an evaluation and development kit for Xilinx Zynq-7000 System on Chip (SoC), which provides periphery for interfacing with additional hardware and storage including USB, Ethernet, and a SD card slot. The dimensions of the board layout are 160 mm × 160 mm. The maximum power consumption is 60 W. The SoC comprises two subsystems namely Processing System (PS) with Advanced Reduced Instruction Set Computer Machine (ARM) Cortex-A9 dual-core processor and Programmable Logic (PL) fabric running at clocks of 666 MHz and 100 MHz, respectively. Thus, this device allows efficient implementation of monitoring and control algorithms by leveraging both advantages of FPGAs for fast signal processing and flexibility of software programmable devices to implement higher level sequence control and communication interfaces. For data acquisition, Analog Devices AD7961 is used enabling AD conversion at a sampling rate of 5 MHz with a resolution of 16 bit. The chip is mounted on an evaluation daughterboard and is connected to the device with the FPGA Mezzanine Card (FMC) connector.

**Figure 2:**
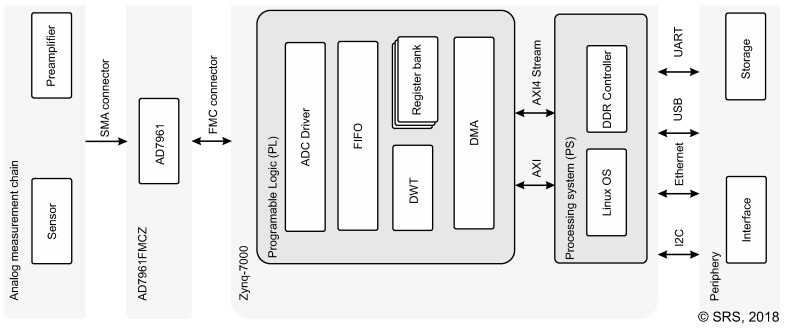
Illustration of the measurement system architecture.

The overall system is illustrated in Figure 2. The PL is used to implement the DWT module, which is used for real-time processing of the raw measurement data. The raw data and DWT coefficients are stored temporarily in a FIFO queue and are transferred afterwards to the PS via Direct Memory Access (DMA) using AXI4-Stream interface. Additionally, maximum, minimum, mean, and energy of the DWT coefficients in each level are stored in the register bank, which is accessed via General Purpose (GP) port. The PS runs a Linux operating system, which is used to implement general functionality of the device. This includes loading drivers and enabling Ethernet at boot time, configuration of the register bank, and control of the data acquisition (start/stop) and storage media. The data path between the PL and PS is made by using High Performance Port (HPF) to achieve low latency and an interrupt line is initialized from the DMA to the PS. After DMA transfer, the raw data and DWT coefficients are read directly from RAM by the PS and stored either on the SD Card or in external memory (e.g., USB drive) in binary format.

### 2.3. Implementation of DWT Module

The wavelet transform is a method for decomposition of non-stationary signals into joint time-frequency domain using an orthogonal basis function, which is referred to as wavelet. In case of the DWT, decomposition of a signal can be achieved by using multirate filter banks, which are constructed from Finite Impulse Response (FIR) filters. Due to decimation of the input signal by passing through each filter bank, the DWT provides a sparse representation of the input signal. Nevertheless, DWT coefficients can yield a perfect reconstruction of the original input signal [27]. Furthermore, time complexity of the algorithm is O(n). Therefore, DWT is well suited for denoising, data compression, and feature extraction in real-time applications.

Different architectures for hardware implementation of the DWT algorithm are proposed including pyramid and polyphase architectures [27]. Regarding sample-wise calculation of DWT coefficients it has to be noted that to ensure suitable reconstruction of the original signal, equalization of delays along all filter paths is required [28].

In Equation (1) the well known implementation of DWT is given as

(1) $$\begin{array}{cc} y_{h}\left[n\right] & =\sum_{k}x\left[n\right]h_{0}\left[k\right]\;\mathrm{and}\\ y_{g}\left[n\right] & =\sum_{k}x\left[n\right]g_{0}\left[k\right], \end{array}$$

where $y_{h}\left[n\right]$ and $y_{g}\left[n\right]$ are the outputs of the high- and low-pass filter, respectively. The related filter coefficients are denoted by $h_{0}\left[k\right]$ and $g_{0}\left[k\right]$. To obtain the DWT coefficients $y_{h}\left[2n\right]$ and $y_{g}\left[2n\right]$, the outputs are decimated by 2. For hardware efficient implementation of DWT, transformations including Noble entities are required to minimize the arithmetic workload and redundancies. Subsequently, polyphase realization using Quadrature Mirror Filter (QMF) pair as described in detail by Cunha et al. [29] is adopted. The main advantage of this approach compared to the classic implementation is the reduction of hardware resources required for synthesis of the algorithm (i.e., adders, multipliers, and number of clock cycles) by a factor of two.

**Figure 3:**
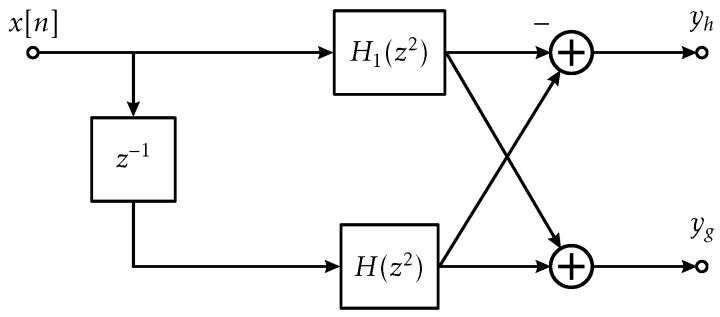
Block diagram of quadrature mirror filter.

Implementation of 1-level DWT is illustrated in Figure 3. Here, x[n] denotes a discrete-time input signal. By using QMF filter bank, the spectrum of x[n] is divided into two sub-bands. Here, $H_{1}\left[z^{2}\right]$ and $H[z^{2}]$ denote the high- and low-pass FIR filters, which are defined by a finite set of coefficients referred to as taps. The number of taps is related to the filter order and the values are determined depending on the related wavelet basis and scaling function. The DWT coefficients are computed sample-wise as the corresponding output of the high- and low-pass filter as

(2) $$\begin{array}{cc} y_{h}\left[2n\right] & =\sum_{k/2}x\left[2n\right]h_{0}\left[2k\right]-\sum_{k/2}x\left[2n+1\right]h_{0}\left[2k+1\right]\;\mathrm{and}\\ y_{g}\left[2n\right] & =\sum_{k/2}x\left[2n\right]h_{0}\left[2k\right]+\sum_{k/2}x\left[2n+1\right]h_{0}\left[2k+1\right]. \end{array}$$

Here, $y_{h}\left[2n\right]$ and $y_{g}\left[2n\right]$ denote the details and approximation coefficients, respectively. Furthermore, $H(z^{2})$ and $H_{1}\left(z^{2}\right)$ in frequency domain correspond to the time domain response $h_{0}\left[2k\right]$ and $h_{0}\left[2k+1\right]$ related to even and odd samples of x[n]. Multilevel DWT can be realized by cascading multiple QMF filter banks. In this case, approximate coefficients are used as input to the subsequent filter bank. The DWT module is implemented using FIR filter with 12 taps and 12-bit quantization using DSP blocks available on the PL. The overall hardware utilization of single level DWT is shown in Table 2. It is worth mentioning that using QMF, only 12 taps are required as compared to 24 taps using the classic implementation.

## 3. Results

To demonstrate the capabilities of the newly developed system for AE measurements and signal processing, experimental results of the application to PLAL are presented in this section. First, correlation between the WD and productivity of PLAL is established. Finally, results of automatic WD adjustment are presented.

### 3.1. Preliminary Investigation of AE Energy

During PLAL, ablation productivity strongly depends on the WD. To realize an automatic adjustment of the WD, a correlation between the AE energy and the productivity of the ablation is established in the sequel. To this end, in-situ UV/VIS spectroscopy and AE measurements were conducted simultaneously at different WD in proximity of the optimal WD using gold targets. Results of the AE measurements are compared to the nanoparticle concentration in the liquid as a reference. During each AE measurement, data are acquired for a duration of 7 s with a sample rate of 4 MHz. Averaged results using a total of 16 individual measurements are reported.

**Figure 4:**
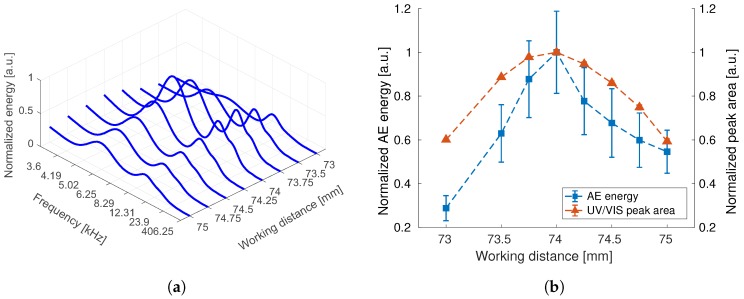
Results for PLAL at different working distances. (**a**) Frequency spectra of AE during ablation (average of 16 measurements); (**b**) Comparison of AE and UV/VIS.

In Figure 4a the frequency spectrum of the AE signal is shown for different WD. At frequencies of 5 kHz and 10 kHz, AE energy peaks are evident. Furthermore, considering peak values of the frequency spectra, dependence of AE intensity on the WD is clear. Maximum energy is obtained for the working distance of 74 mm. In Figure 4b, comparison to in-situ UV/VIS measurements is presented. The maximum concentration of nanoparticles in the liquid is also obtained at a WD of 74 mm. As already reported in [1], a correlation between AE energy and productivity of PLAL can be observed. In difference to the work of Zhu et al. [1], a piezoelectric, mechanically coupled sensor is used, which has higher frequency bandwidth compared to a microphone. However, strong scatter of the AE energy values is evident as it can be seen in Figure 4. Thus, approximate coefficients of DWT are used to reduce noise.

### 3.2. Automatic Adjustment of Working Distance

During PLAL, maximum productivity is achieved if the WD is adjusted so that the target surface is placed close to the focal point of the laser. However, the exact position leading to the best possible productivity is not known [15]. Furthermore, the corresponding spindle position is subject to variability, which is related to the mounting of the ablation chamber on the translatory stage and the placement of the target in the ablation chamber. Therefore, tuning of the optimal WD is difficult and time consuming so that due to the physical nature of the process, PLAL is frequently performed at possibly suboptimal WD.

To find and maintain a suitable WD, an iterative search algorithm for automatic positioning of the ablation chamber, which uses AE energy as objective, is realized. Here, a gradient-based search heuristic, which does not require a mathematical model, is used. The raw measurement data is processed by the DWT module in real-time using the PL core. The search algorithm is implemented on the PS running within the Linux OS. To reduce noise, AE energy is obtained from the approximate coefficients of the DWT. As it is shown in the previous section, the AE energy is a convex function of the position so the optimal WD is expected at the maximum AE energy. Within each step, the position is changed in search direction with a fixed step size and measurement of the related AE is conducted. However, due to scatter of the AE energy, direct calculation of the gradient from two consecutive measurements is not feasible. Therefore, AE energy obtained at each step is stored in a buffer of fixed size holding the past *N* values. The gradient is estimated by an averaging procedure. Additionally, after each change of the search direction, a minimum number of measurements must be acquired. Moving towards the desired position, a positive gradient of the AE energy is obtained. If the estimated gradient is negative, the search direction is reversed. Parameters of the search algorithm such as step size and the buffer size can be determined empirically.

Subsequently, experimental results of the automatic positioning algorithm on the PLAL test rig are presented. Each experiment was run for approximately 30 min. Different parameters for step size and buffer size were tested. During each iteration, full AE waveform data are acquired for 1.5 s at a sample rate of 5 MHz. The real-time DWT module is used to filter the AE signal. After 30 min, the automatic positioning was stopped to verify the determined position by manual tuning. Because the WD is dependent on the mounting of the ablation chamber on the translational stage and the placement of the target, WD can not be determined accurately and hence spindle position is reported, which is directly related to the WD.

**Figure 5:**
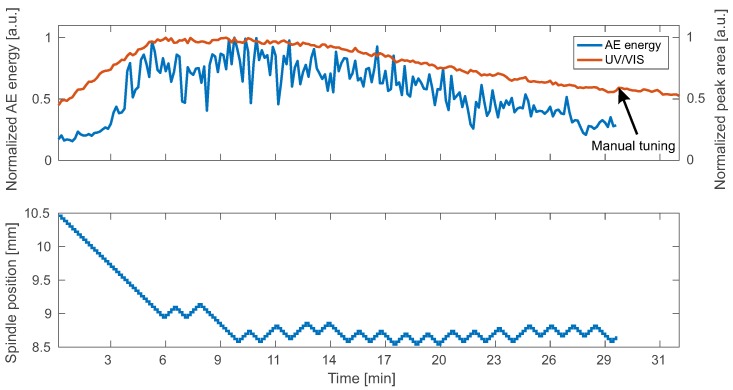
Automatic positioning results: experimental run I.

**Figure 6:**
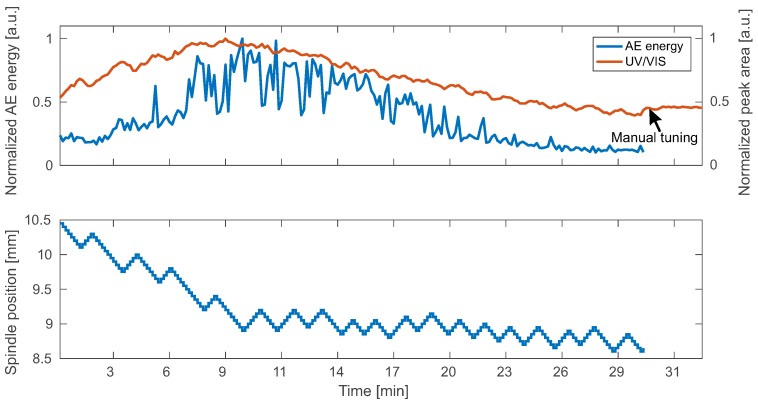
Automatic positioning results: experimental run II.

Results of two independent runs are presented in Figure 5 and Figure 6. Here, AE energy and UV/VIS measurements are in good agreement. Furthermore, the related spindle position is shown. The initial position was 10.5 mm in both cases. In the beginning of the experiment, the spindle position is adjusted towards the optimal WD by the algorithm. At the same time, the rise in AE energy and UV/VIS measurements indicates increased productivity. After 10 min, the spindle position settles in a range between 8.5 mm and 9 mm. After a period of constant productivity, decay of the AE energy is observed while the optimal WD does not change.

## 4. Summary and Conclusions

Regarding SHM, AE analysis is frequently used for highly sensitive in-situ inspection. Promising results have been reported in different applications such as process monitoring and control, where AE is suggested as highly sensitive in-situ measurement technique. However, most of the analyses are carried out offline due to the complexity of signal processing algorithms (e.g., feature extraction and classification). To provide timely statements regarding the current system state or to realize related control actions, online processing of AE is required. In this work, a suitable low-cost solution is suggested. Therefore, a hardware architecture is proposed, which is particularly suited for embedded implementations due to its small form factor and low power consumption. At the example of PLAL nanoparticle production, it is shown that full waveform data from the AE sensor can be acquired and processed at a sample rate of 5 MHz. Also, joint time-frequency domain representation of the measurement signal is available in real-time using FPGA-based implementation of DWT, which is used to reduce noise. Alternatively, DWT coefficients can also be used as features in a classification scheme to be implemented for AE measurements. A sample rate of 5 MHz is achieved at 16 bit resolution. Finally, an application to process control is demonstrated. Results of the AE measurements are used to calculate the control input for guidance of the PLAL process in a timely manner. This implies an important contribution towards implementation of AE-based SHM systems.

As a proof-of-concept, automatic adjustment of the Working Distance (WD) during PLAL for nanoparticle production is used. In the field of PLAL, the adjustment of a suitable WD is important but difficult and time consuming. Therefore, in-situ measurement techniques are necessary to assess the productivity and to implement automatic adjustment of the WD. Typically, UV/VIS spectroscopy is used to determine the concentration of nanoparticles in liquids. However, this technique can not be applied in general due to the dependence of the signal on the material, the particle size, and the particle shape. In addition, online UV/VIS spectroscopy is limited to a small concentration range. The use of AE measurements is a new approach to in-situ characterization of PLAL productivity. Generally, a good correlation between UV/VIS and AE energy is observed using copper and gold targets. It can be concluded that AE measurements provide a suitable means to assess the productivity of PLAL.

However, compared to UV/VIS measurements, large scatter of the AE energy is observed, which makes automatic positioning difficult. This is possibly related to the nonstationary character of the process, which until now is not perfectly understood. Possible explanations are absorption and scattering of the laser energy by cavitation bubbles and evaporated liquid inside of the ablation chamber as well as effects related to pulsating liquid flow (plunger pump) and the scan pattern. Also, at a given WD the productivity of PLAL in a flow setup is expected to be constant due to the continuous removal of the nanoparticles from the ablation zone. The decay of UV/VIS and AE energy (and thus productivity), which is observed during the experiments, could be attributed to changed process dynamics due to thermal effects i.e., heating of the target and the ablation chamber. The experimental results show that using AE measurements, close to optimal WD with an accuracy between 0.25 mm and 0.75 mm can be achieved by applying a typical search algorithm.

## Figures and Tables

**Table 1:** Equipment of PLAL test rig.

Equipment	Specification
Laser: Rofin Sinar RS-Marker 100D	Wavelength: 1064 nm Power: 32.5 W Repetition rate: 5 kHz Pulse duration: 40 ns Scan speed: 600 mm/s
Plunger pump: Ismatec RHP 100994	Flow rate: 50 mL/min
UV/VIS	Lamp: Ocean Optics DH-Mini Detector: Red-Tide USB 650

**Table 2:** Hardware utilization of 1-level DWT module.

BRAM	DSP48E1	LUT	FF
116.5 (83.21 %)	12 (5.45 %)	7536 (14.17 %)	9274 (8.72 %)
